# Revealing divergent evolution, identifying circular permutations and detecting active-sites by protein structure comparison

**DOI:** 10.1186/1472-6807-6-18

**Published:** 2006-09-02

**Authors:** Luonan Chen, Ling-Yun Wu, Yong Wang, Shihua Zhang, Xiang-Sun Zhang

**Affiliations:** 1 Institute of Systems Biology, Shanghai University, Shanghai 200444, China; 2 Osaka Sangyo University, Nakagaito 3-1-1, Daito, Osaka 574-8530, Japan; 3 Institute of Applied Mathematics, Academy of Mathematics and Systems Science, CAS, Beijing 100080, China; 4 Graduate School of the Chinese Academy of Sciences, Beijing 100049,China

## Abstract

**Background:**

Protein structure comparison is one of the most important problems in computational biology and plays a key role in protein structure prediction, fold family classification, motif finding, phylogenetic tree reconstruction and protein docking.

**Results:**

We propose a novel method to compare the protein structures in an accurate and efficient manner. Such a method can be used to not only reveal divergent evolution, but also identify circular permutations and further detect active-sites. Specifically, we define the structure alignment as a multi-objective optimization problem, i.e., maximizing the number of aligned atoms and minimizing their root mean square distance. By controlling a single distance-related parameter, theoretically we can obtain a variety of optimal alignments corresponding to different optimal matching patterns, i.e., from a large matching portion to a small matching portion. The number of variables in our algorithm increases with the number of atoms of protein pairs in almost a linear manner. In addition to solid theoretical background, numerical experiments demonstrated significant improvement of our approach over the existing methods in terms of quality and efficiency. In particular, we show that divergent evolution, circular permutations and active-sites (or structural motifs) can be identified by our method. The software SAMO is available upon request from the authors, or from  and .

**Conclusion:**

A novel formulation is proposed to accurately align protein structures in the framework of multi-objective optimization, based on a sequence order-independent strategy. A fast and accurate algorithm based on the bipartite matching algorithm is developed by exploiting the special features. Convergence of computation is shown in experiments and is also theoretically proven.

## Background

Proteins are macromolecules that regulate all biological processes in a living organism, and their structures are generally better conserved than sequences. Thus, identifying similarity of structures by comparing proteins could yield valuable clues to their function, and can be employed to fold family classification, motif finding, phylogenetic tree reconstruction and even protein docking. So far, many algorithms have been developed for the structure alignment problem [1-3] including distance-based methods and vector-based methods, such as iterative dynamical programming [4,5], fuzzy matching method [6], mean field equation approximation [7,8], distance matrix alignment method Dali [9], combinatorial extension method CE [10] and genetic algorithm [11]. Despite the relative success, there is much room for improvement in terms of quality and computational efficiency of the alignment [3,12]. On the other hand, from the viewpoint of optimization, there are two criteria for distance-based algorithms of structure comparison, i.e., maximizing the number of the aligned atoms and minimizing the matching distance between two protein's aligned atoms. Such two objectives clearly have a trade-off relationship [7], i.e., minimizing the matching distance usually leads to decrease of the number of aligned atoms whereas maximizing the number of aligned atoms will lead large matching distance. In other words, the solutions of such an alignment problem form a Pareto set [13].

With this clue, this paper presents a novel method in the framework of multi-objective optimization [13], which is called SAMO (protein Structure Alignment tool based on Multiple Objective optimization). We define the structure alignment as a two-objective optimization problem with both discrete and continuous variables, i.e., maximizing the number of aligned atoms and minimizing their root mean square distance (RMSD) in the same time. The discrete variables represent matching relation between atoms whereas the continuous variables include a translation vector and a rotation matrix with which one protein matches the other as a rigid body. In particular, in contrast to the conventional methods, we adopt a sequence order-independent strategy in the formulation of structure alignment problem. This allows us to detect similarity between proteins in a more general way, e.g. revealing divergent evolution, detecting circular permutations and identifying active-sites (or structural motifs). In other words, the similarity can be found not only between homologous structures but also between active sites of convergent structures, between different folding motifs, between the scaffolds of unrelated proteins and between recurring stable configurations in the interior of proteins. As shown in this paper, we succeeded in finding the similarity of divergently evolved proteins as well as that of convergent proteins [14].

Although a pairwise protein comparison can theoretically be formulated as a multi-objective optimization problem, numerically it is still a complicated computational problem, in particular for the comparison of large-size proteins. To alleviate such computation burden, we develop a decomposition technique to divide the original problem into two subproblems by exploiting the special features of the protein alignment problem, i.e., one linear programming subproblem (LPS) for the atom matching and one weighted least square subproblem (LSS) for coordinate transformation. A very efficient bipartite matching algorithm is proposed for optimizing the LPS, whereas the LSS is solved by the singular value decomposition (SVD) technique. By controlling a single distance-related parameter, theoretically we can obtain a variety of optimal alignments corresponding to different optimal matching patterns, which all belong to the Pareto set. In other words, depending on how close we require to match a pair of proteins, we can obtain a set of optimal alignment solution, from a large portion matching to a small portion matching. The main features for this paper are summarized as follows.

• We propose a novel formulation to align protein structures, reveal divergent evolution, detect circular permutations and identify structural motifs in the framework of multi-objective optimization.

• We develop an efficient and accurate algorithm based on bipartite matching algorithm to solve the multi-objective programming, and the convergence of the algorithm is also theoretically guaranteed.

Although our algorithm can obtain an optimal alignment, the resulting solution may not be globally optimal due to the non-convexity of the protein structure alignment problem. Generally, it is well known that the annealing technique is effective to alleviate the influence of initial conditions on the solution. This paper adopts an annealing procedure for expanding the searching region to improve quality of solution. Other features of the model include: according to information of the matching matrix, the algorithm has the ability to identify circular permutations [7,8] and active sites; no heuristic parameter, such as gap penalty, is required in our formulation. To demonstrate the proposed method, we use several benchmark examples [7,10,6] from Protein Data Bank as well as SCOP database for numerical simulation. In addition to solid theoretical background, numerical experiments show significant improvement of our approach over the existing methods in terms of both quality and efficiency.

## Implementation

The method presented in this paper is mainly based on the preliminary version in [15]. In this section, we formulate the pairwise structure alignment problem as a multi-objective optimization problem with the similar notation to that of [8,15].

### Preliminaries

Define *n*_*x *_and *n*_*y *_to be the number of atoms of two proteins *X *= (*X*_1_, ..., Xnx
 MathType@MTEF@5@5@+=feaafiart1ev1aaatCvAUfKttLearuWrP9MDH5MBPbIqV92AaeXatLxBI9gBaebbnrfifHhDYfgasaacH8akY=wiFfYdH8Gipec8Eeeu0xXdbba9frFj0=OqFfea0dXdd9vqai=hGuQ8kuc9pgc9s8qqaq=dirpe0xb9q8qiLsFr0=vr0=vr0dc8meaabaqaciaacaGaaeqabaqabeGadaaakeaacqWGybawdaWgaaWcbaGaemOBa42aaSbaaWqaaiabdIha4bqabaaaleqaaaaa@3127@) and *Y *= (*Y*_1_, ..., Yny
 MathType@MTEF@5@5@+=feaafiart1ev1aaatCvAUfKttLearuWrP9MDH5MBPbIqV92AaeXatLxBI9gBaebbnrfifHhDYfgasaacH8akY=wiFfYdH8Gipec8Eeeu0xXdbba9frFj0=OqFfea0dXdd9vqai=hGuQ8kuc9pgc9s8qqaq=dirpe0xb9q8qiLsFr0=vr0=vr0dc8meaabaqaciaacaGaaeqabaqabeGadaaakeaacqWGzbqwdaWgaaWcbaGaemOBa42aaSbaaWqaaiabdMha5bqabaaaleqaaaaa@312B@), where *X*_*i *_= (*x*_*i*,1_, *x*_*i*,2_, *x*_*i*,3_) and *Y*_*j *_= (*y*_*j*,1_, *y*_*j*,2_, *y*_*j*,3_) ∈ **R**^3 ^(*i *= 1, ..., *n*_*x*_; *j *= 1, ..., *n*_*y*_) are the atom coordinates, which correspond to *C*_*α *_or *C*_*β *_atoms along the backbones. A square distance metric between the chain atoms is adopted, i.e. dij2=|Xi−Yj|2=∑k=13(xi,k−yj,k)2
 MathType@MTEF@5@5@+=feaafiart1ev1aaatCvAUfKttLearuWrP9MDH5MBPbIqV92AaeXatLxBI9gBaebbnrfifHhDYfgasaacH8akY=wiFfYdH8Gipec8Eeeu0xXdbba9frFj0=OqFfea0dXdd9vqai=hGuQ8kuc9pgc9s8qqaq=dirpe0xb9q8qiLsFr0=vr0=vr0dc8meaabaqaciaacaGaaeqabaqabeGadaaakeaacqWGKbazdaqhaaWcbaGaemyAaKMaemOAaOgabaGaeGOmaidaaOGaeyypa0ZaaqWaceaacqWGybawdaWgaaWcbaGaemyAaKgabeaakiabgkHiTiabdMfaznaaBaaaleaacqWGQbGAaeqaaaGccaGLhWUaayjcSdWaaWbaaSqabeaacqaIYaGmaaGccqGH9aqpdaaeWaqaamaabmGabaGaemiEaG3aaSbaaSqaaiabdMgaPjabcYcaSiabdUgaRbqabaGccqGHsislcqWG5bqEdaWgaaWcbaGaemOAaOMaeiilaWIaem4AaSgabeaaaOGaayjkaiaawMcaamaaCaaaleqabaGaeGOmaidaaaqaaiabdUgaRjabg2da9iabigdaXaqaaiabiodaZaqdcqGHris5aaaa@5329@ is the square distance between the atom *i *in *X *and the atom *j *in *Y*. We view each protein chain as a rigid geometric body in this paper. The coordinate transformation of a rigid body is generally expressed by a translation vector *A *∈ **R**^3 ^and a rotation matrix *R *∈ **R**^3 × 3^, i.e., X^i
 MathType@MTEF@5@5@+=feaafiart1ev1aaatCvAUfKttLearuWrP9MDH5MBPbIqV92AaeXatLxBI9gBaebbnrfifHhDYfgasaacH8akY=wiFfYdH8Gipec8Eeeu0xXdbba9frFj0=OqFfea0dXdd9vqai=hGuQ8kuc9pgc9s8qqaq=dirpe0xb9q8qiLsFr0=vr0=vr0dc8meaabaqaciaacaGaaeqabaqabeGadaaakeaacuWGybawgaqcamaaBaaaleaacqWGPbqAaeqaaaaa@2F7C@ = *A *+ *RX*_*i *_for the atom *i *of the chain *X*, where there are six independent variables for the translation vector and the rotation matrix due to the rigid body transformation. For a pairwise structure alignment, we fix the coordinates of *Y*, which is assumed to be longer than *X*. Therefore, after coordinate transformation, a square distance between the atom *i *in *X *and the atom *j *in *Y *is defined as follows

dij2
 MathType@MTEF@5@5@+=feaafiart1ev1aaatCvAUfKttLearuWrP9MDH5MBPbIqV92AaeXatLxBI9gBaebbnrfifHhDYfgasaacH8akY=wiFfYdH8Gipec8Eeeu0xXdbba9frFj0=OqFfea0dXdd9vqai=hGuQ8kuc9pgc9s8qqaq=dirpe0xb9q8qiLsFr0=vr0=vr0dc8meaabaqaciaacaGaaeqabaqabeGadaaakeaacqWGKbazdaqhaaWcbaGaemyAaKMaemOAaOgabaGaeGOmaidaaaaa@31D4@ = |*A *+ *RX*_*i *_- *Y*_*j*_|^2 ^    (1)

A matching matrix *S *with binary elements *s*_*ij *_is defined to describe matching of two atoms for *i *= 1, ..., *n*_*x*_; *j *= 1, ..., *n*_*y*_:

sij={1if atom i in the chain X matches atom j in the chain Y0otherwise     (2)
 MathType@MTEF@5@5@+=feaafiart1ev1aaatCvAUfKttLearuWrP9MDH5MBPbIqV92AaeXatLxBI9gBaebbnrfifHhDYfgasaacH8akY=wiFfYdH8Gipec8Eeeu0xXdbba9frFj0=OqFfea0dXdd9vqai=hGuQ8kuc9pgc9s8qqaq=dirpe0xb9q8qiLsFr0=vr0=vr0dc8meaabaqaciaacaGaaeqabaqabeGadaaakeaacqWGZbWCdaWgaaWcbaGaemyAaKMaemOAaOgabeaakiabg2da9maaceqabaqbaeaabiGaaaqaaiabigdaXaqaaiabbMgaPjabbAgaMjabbccaGiabbggaHjabbsha0jabb+gaVjabb2gaTjabbccaGiabdMgaPjabbccaGiabbMgaPjabb6gaUjabbccaGiabbsha0jabbIgaOjabbwgaLjabbccaGiabbogaJjabbIgaOjabbggaHjabbMgaPjabb6gaUjabbccaGiabdIfayjabbccaGiabb2gaTjabbggaHjabbsha0jabbogaJjabbIgaOjabbwgaLjabbohaZjabbccaGiabbggaHjabbsha0jabb+gaVjabb2gaTjabbccaGiabdQgaQjabbccaGiabbMgaPjabb6gaUjabbccaGiabbsha0jabbIgaOjabbwgaLjabbccaGiabbogaJjabbIgaOjabbggaHjabbMgaPjabb6gaUjabbccaGiabdMfazbqaaiabicdaWaqaaiabb+gaVjabbsha0jabbIgaOjabbwgaLjabbkhaYjabbEha3jabbMgaPjabbohaZjabbwgaLbaaaiaawUhaaiaaxMaacaWLjaWaaeWaceaacqaIYaGmaiaawIcacaGLPaaaaaa@86AD@

Clearly *S *is an *n*_*x *_× *n*_*y *_matrix with only binary elements. With allowing existence of gaps, each atom in one chain must match at most one atom in the other. Therefore, we have the following conditions.

∑i=1nxsij≤1 for j=1,...,ny     (3)
 MathType@MTEF@5@5@+=feaafiart1ev1aaatCvAUfKttLearuWrP9MDH5MBPbIqV92AaeXatLxBI9gBaebbnrfifHhDYfgasaacH8akY=wiFfYdH8Gipec8Eeeu0xXdbba9frFj0=OqFfea0dXdd9vqai=hGuQ8kuc9pgc9s8qqaq=dirpe0xb9q8qiLsFr0=vr0=vr0dc8meaabaqaciaacaGaaeqabaqabeGadaaakeaadaaeWbqaaiabdohaZnaaBaaaleaacqWGPbqAcqWGQbGAaeqaaaqaaiabdMgaPjabg2da9iabigdaXaqaaiabd6gaUnaaBaaameaacqWG4baEaeqaaaqdcqGHris5aOGaeyizImQaeGymaeJaeeiiaaIaeeOzayMaee4Ba8MaeeOCaiNaeeiiaaIaemOAaOMaeyypa0JaeGymaeJaeiilaWIaeiOla4IaeiOla4IaeiOla4IaeiilaWIaemOBa42aaSbaaSqaaiabdMha5bqabaGccaWLjaGaaCzcamaabmGabaGaeG4mamdacaGLOaGaayzkaaaaaa@508A@

∑j=1nysij≤1 for i=1,...,nx     (4)
 MathType@MTEF@5@5@+=feaafiart1ev1aaatCvAUfKttLearuWrP9MDH5MBPbIqV92AaeXatLxBI9gBaebbnrfifHhDYfgasaacH8akY=wiFfYdH8Gipec8Eeeu0xXdbba9frFj0=OqFfea0dXdd9vqai=hGuQ8kuc9pgc9s8qqaq=dirpe0xb9q8qiLsFr0=vr0=vr0dc8meaabaqaciaacaGaaeqabaqabeGadaaakeaadaaeWbqaaiabdohaZnaaBaaaleaacqWGPbqAcqWGQbGAaeqaaaqaaiabdQgaQjabg2da9iabigdaXaqaaiabd6gaUnaaBaaameaacqWG5bqEaeqaaaqdcqGHris5aOGaeyizImQaeGymaeJaeeiiaaIaeeOzayMaee4Ba8MaeeOCaiNaeeiiaaIaemyAaKMaeyypa0JaeGymaeJaeiilaWIaeiOla4IaeiOla4IaeiOla4IaeiilaWIaemOBa42aaSbaaSqaaiabdIha4bqabaGccaWLjaGaaCzcamaabmGabaGaeGinaqdacaGLOaGaayzkaaaaaa@508C@

**Figure 1:**
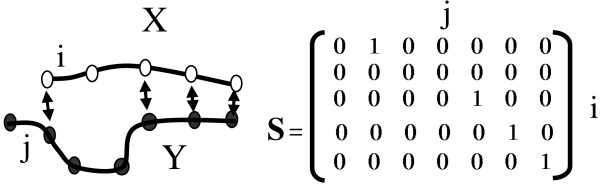
An example for two protein chains and their assignment matrix *S *with *n*_*x *_= 5 and *n*_*y *_= 7.

We show a simple example in Figure 1 to illustrate a matching matrix *S *with *n*_*x *_= 5 and *n*_*y *_= 7, where a row or a column with all zeros means a gap. Then, the total square distances *T *and the total number *m *for the aligned atoms between the two proteins are respectively expressed as:

T(S,A,R)=∑i=1nx∑j=1nysij|A+RXi−Yj|2     (5)
 MathType@MTEF@5@5@+=feaafiart1ev1aaatCvAUfKttLearuWrP9MDH5MBPbIqV92AaeXatLxBI9gBaebbnrfifHhDYfgasaacH8akY=wiFfYdH8Gipec8Eeeu0xXdbba9frFj0=OqFfea0dXdd9vqai=hGuQ8kuc9pgc9s8qqaq=dirpe0xb9q8qiLsFr0=vr0=vr0dc8meaabaqaciaacaGaaeqabaqabeGadaaakeaacqWGubavcqGGOaakcqWGtbWucqGGSaalcqWGbbqqcqGGSaalcqWGsbGucqGGPaqkcqGH9aqpdaaeWbqaamaaqahabaGaem4Cam3aaSbaaSqaaiabdMgaPjabdQgaQbqabaaabaGaemOAaOMaeyypa0JaeGymaedabaGaemOBa42aaSbaaWqaaiabdMha5bqabaaaniabggHiLdaaleaacqWGPbqAcqGH9aqpcqaIXaqmaeaacqWGUbGBdaWgaaadbaGaemiEaGhabeaaa0GaeyyeIuoakmaaemGabaGaemyqaeKaey4kaSIaemOuaiLaemiwaG1aaSbaaSqaaiabdMgaPbqabaGccqGHsislcqWGzbqwdaWgaaWcbaGaemOAaOgabeaaaOGaay5bSlaawIa7amaaCaaaleqabaGaeGOmaidaaOGaaCzcaiaaxMaadaqadiqaaiabiwda1aGaayjkaiaawMcaaaaa@5D01@

m(S)=∑i=1nx∑j=1nysij     (6)
 MathType@MTEF@5@5@+=feaafiart1ev1aaatCvAUfKttLearuWrP9MDH5MBPbIqV92AaeXatLxBI9gBaebbnrfifHhDYfgasaacH8akY=wiFfYdH8Gipec8Eeeu0xXdbba9frFj0=OqFfea0dXdd9vqai=hGuQ8kuc9pgc9s8qqaq=dirpe0xb9q8qiLsFr0=vr0=vr0dc8meaabaqaciaacaGaaeqabaqabeGadaaakeaacqWGTbqBcqGGOaakcqWGtbWucqGGPaqkcqGH9aqpdaaeWbqaamaaqahabaGaem4Cam3aaSbaaSqaaiabdMgaPjabdQgaQbqabaaabaGaemOAaOMaeyypa0JaeGymaedabaGaemOBa42aaSbaaWqaaiabdMha5bqabaaaniabggHiLdaaleaacqWGPbqAcqGH9aqpcqaIXaqmaeaacqWGUbGBdaWgaaadbaGaemiEaGhabeaaa0GaeyyeIuoakiaaxMaacaWLjaWaaeWaceaacqaI2aGnaiaawIcacaGLPaaaaaa@4B51@

### Multi-objective optimization for structure comparison

For structure alignment problem, there is generally a trade-off relation [7,8] between the distance and the number of aligned atoms. Therefore, a pairwise structure alignment problem can be formulated as a two-objective optimization problem [13] with discrete variables *S *and continuous variables (*A*, *R*):

minimize *T*(*S*, *A*, *R*)     for *S*, *A*, *R *    (7)

maximize *m*(*S*)

subject to (3)–(4)     *s*_*ij*_∈ {0,1}

where the first objective is to minimize the total square distances of the aligned atoms, and the second objective is to maximize the total number of aligned atoms for the two proteins. Notice that there is no heuristic gap penalty term in the formulation.

All of the optimal solutions for the two-objective optimization problem form a Pareto set [13]. The problem can be solved by transforming the two objectives of (7) into a single objective. One typical technique is the *ε*-method [13], which alternates a positive scalar parameter *λ *to obtain the Pareto set, with the following formulation.

minimize ∑i=1nx∑j=1nysij(|A+RXi−Yj|2−λ2)for S,A,Rsubject to (3)−(4)sij∈{0,1}     (8)
 MathType@MTEF@5@5@+=feaafiart1ev1aaatCvAUfKttLearuWrP9MDH5MBPbIqV92AaeXatLxBI9gBaebbnrfifHhDYfgasaacH8akY=wiFfYdH8Gipec8Eeeu0xXdbba9frFj0=OqFfea0dXdd9vqai=hGuQ8kuc9pgc9s8qqaq=dirpe0xb9q8qiLsFr0=vr0=vr0dc8meaabaqaciaacaGaaeqabaqabeGadaaakeaafaqaaeGacaaabaGaeeyBa0MaeeyAaKMaeeOBa4MaeeyAaKMaeeyBa0MaeeyAaKMaeeOEaONaeeyzauMaeeiiaaYaaabCaeaadaaeWbqaaiabdohaZnaaBaaaleaacqWGPbqAcqWGQbGAaeqaaaqaaiabdQgaQjabg2da9iabigdaXaqaaiabd6gaUnaaBaaameaacqWG5bqEaeqaaaqdcqGHris5aaWcbaGaemyAaKMaeyypa0JaeGymaedabaGaemOBa42aaSbaaWqaaiabdIha4bqabaaaniabggHiLdGcdaqadiqaamaaemGabaGaemyqaeKaey4kaSIaemOuaiLaemiwaG1aaSbaaSqaaiabdMgaPbqabaGccqGHsislcqWGzbqwdaWgaaWcbaGaemOAaOgabeaaaOGaay5bSlaawIa7amaaCaaaleqabaGaeGOmaidaaOGaeyOeI0ccciGae83UdW2aaWbaaSqabeaacqaIYaGmaaaakiaawIcacaGLPaaaaeaacqqGMbGzcqqGVbWBcqqGYbGCcqqGGaaicqWGtbWucqGGSaalcqWGbbqqcqGGSaalcqWGsbGuaeaacqqGZbWCcqqG1bqDcqqGIbGycqqGQbGAcqqGLbqzcqqGJbWycqqG0baDcqqGGaaicqqG0baDcqqGVbWBcqqGGaaidaqadiqaaiabiodaZaGaayjkaiaawMcaaiabgkHiTmaabmGabaGaeGinaqdacaGLOaGaayzkaaaabaGaem4Cam3aaSbaaSqaaiabdMgaPjabdQgaQbqabaGccqGHiiIZdaGadeqaaiabicdaWiabcYcaSiabigdaXaGaay5Eaiaaw2haaaaacaWLjaGaaCzcamaabmGabaGaeGioaGdacaGLOaGaayzkaaaaaa@8DE7@

From the definition of *T*(*S*, *A*, *R*) and *m*(*S*), the objective is minimizing *T*(*S*, *A*, *R*) - *λ*^2^*m*(*S*). We can theoretically obtain all optimal solutions belonging to the Pareto set, by changing the parameter *λ *in the single-objective optimization problem (8). Clearly, *λ *transforms the number *m *into equivalent square distance, and controls the balance of *T *and *m *[15]. It should be noted that |*A *+ *RX*_*i *_- *Y*_*j*_|^2 ^- *λ*^2 ^= dij2
 MathType@MTEF@5@5@+=feaafiart1ev1aaatCvAUfKttLearuWrP9MDH5MBPbIqV92AaeXatLxBI9gBaebbnrfifHhDYfgasaacH8akY=wiFfYdH8Gipec8Eeeu0xXdbba9frFj0=OqFfea0dXdd9vqai=hGuQ8kuc9pgc9s8qqaq=dirpe0xb9q8qiLsFr0=vr0=vr0dc8meaabaqaciaacaGaaeqabaqabeGadaaakeaacqWGKbazdaqhaaWcbaGaemyAaKMaemOAaOgabaGaeGOmaidaaaaa@31D4@ - *λ*^2 ^implies that *λ *has the same physical meaning and scale as the distance of *d*_*ij*_. This paper exploits this property to drastically simplify the computation of the problem. If *λ *is small, the optimal alignment has a smaller set of aligned atoms (*m*) but with a tight matching (*T*). In contrast, for a big *λ*, we can have a bigger set of aligned atoms but with a rough matching. Therefore, rather than one solution, we can obtain a set of optimal solutions for different pairs of (*T*, *m*) by changing *λ*. In addition to the accurate form without any heuristic parameters of gaps in the model, the objective function is a linear form of *S*, and in formulation (8) the number *m *directly pairs with the square distance *T*. Comparing SAMO with the conventional superimposition-alignment approach, such as iterative dynamic programming [4], there are mainly two differences. One is that many of the conventional superimposition-alignment approaches use the heuristic objective function (e.g. using heuristic parameters in the similarity criterion) or the heuristic gap penalty terms in the formulation, which cause not only the poor quality of alignment but also the poor convergence. Another one is about the searching space, which usually is locally restricted depending on the coordinate transformation or superimposition strategy for the conventional methods, whereas our algorithm theoretically has the ability for implicit complete exploration of the entire space of alignments.

### Decomposing structure comparison problem

We exploit the special features of the formulation to decompose the optimization problem of the protein structure comparison. Clearly, (8) is a mixed integer programming for a given *λ *and has a special structure, i.e., all of the terms in the constraints (3)–(4) are not related to the continuous variables (*A*, *R*). Because of such a special feature, (8) can be decomposed into two subproblems, i.e., a weighted least square subproblem (LSS) that is to find the best transformation of coordinates for the protein *X*, and an integer linear programming subproblem (LPS) that is to find the best superposition for the protein pairs. The procedure of SAMO is an iterative computation of LSS and LPS in succession. Next, we give detail explanation for each subproblem and the solving technique.

### Weighted Least Square Subproblem (LSS)

(9) is the LSS for solving (*A*, *R*) with fixed (*S*, *λ*)

minimize ∑i=1nx∑j=1nysij|A+RXi−Yj|2     (9)
 MathType@MTEF@5@5@+=feaafiart1ev1aaatCvAUfKttLearuWrP9MDH5MBPbIqV92AaeXatLxBI9gBaebbnrfifHhDYfgasaacH8akY=wiFfYdH8Gipec8Eeeu0xXdbba9frFj0=OqFfea0dXdd9vqai=hGuQ8kuc9pgc9s8qqaq=dirpe0xb9q8qiLsFr0=vr0=vr0dc8meaabaqaciaacaGaaeqabaqabeGadaaakeaacqqGTbqBcqqGPbqAcqqGUbGBcqqGPbqAcqqGTbqBcqqGPbqAcqqG6bGEcqqGLbqzcqqGGaaidaaeWbqaamaaqahabaGaem4Cam3aaSbaaSqaaiabdMgaPjabdQgaQbqabaaabaGaemOAaOMaeyypa0JaeGymaedabaGaemOBa42aaSbaaWqaaiabdMha5bqabaaaniabggHiLdaaleaacqWGPbqAcqGH9aqpcqaIXaqmaeaacqWGUbGBdaWgaaadbaGaemiEaGhabeaaa0GaeyyeIuoakmaaemGabaGaemyqaeKaey4kaSIaemOuaiLaemiwaG1aaSbaaSqaaiabdMgaPbqabaGccqGHsislcqWGzbqwdaWgaaWcbaGaemOAaOgabeaaaOGaay5bSlaawIa7amaaCaaaleqabaGaeGOmaidaaOGaaCzcaiaaxMaadaqadiqaaiabiMda5aGaayjkaiaawMcaaaaa@5FBC@

which is a weighted least square problem of two 3-D chains and can actually be solved analytically [7,6,16]. Notice that for the LSS, in addition to (3)–(4), λ2∑i=1nx∑j=1nysij
 MathType@MTEF@5@5@+=feaafiart1ev1aaatCvAUfKttLearuWrP9MDH5MBPbIqV92AaeXatLxBI9gBaebbnrfifHhDYfgasaacH8akY=wiFfYdH8Gipec8Eeeu0xXdbba9frFj0=OqFfea0dXdd9vqai=hGuQ8kuc9pgc9s8qqaq=dirpe0xb9q8qiLsFr0=vr0=vr0dc8meaabaqaciaacaGaaeqabaqabeGadaaakeaaiiGacqWF7oaBdaahaaWcbeqaaiabikdaYaaakmaaqadabaWaaabmaeaacqWGZbWCdaWgaaWcbaGaemyAaKMaemOAaOgabeaaaeaacqWGQbGAcqGH9aqpcqaIXaqmaeaacqWGUbGBdaWgaaadbaGaemyEaKhabeaaa0GaeyyeIuoaaSqaaiabdMgaPjabg2da9iabigdaXaqaaiabd6gaUnaaBaaameaacqWG4baEaeqaaaqdcqGHris5aaaa@4498@ is constant due to the fixed (*S*, *λ*), which has no effect on the optimization and is eliminated from the objective function in (8). Numerically, *R *and *A *can also be obtained by singular value decomposition (SVD) as shown in Appendix A.1 of [7]. There are six independent variables for LSS. LSS pulls the protein *X *closer to the protein *Y *by computing the optimal rotation matrix *R *and translation vector *A*. Note that LSS is not affected by those coordinate pairs (*X*_*i*_, *Y*_*j*_) with *s*_*ij *_= 0, which are actually known before the computation of each iteration. Such a property is exploited in next section to drastically improve the efficiency of LPS computation.

### Integer Linear Programming Subproblem (LPS)

(10) is the LPS for solving *S *with fixed (*A*, *R*, *λ*)

maximize −∑i=1nx∑j=1nysij(|A+RXi−Yj|2−λ2)subject to (3)−(4)sij∈{0,1}     (10)
 MathType@MTEF@5@5@+=feaafiart1ev1aaatCvAUfKttLearuWrP9MDH5MBPbIqV92AaeXatLxBI9gBaebbnrfifHhDYfgasaacH8akY=wiFfYdH8Gipec8Eeeu0xXdbba9frFj0=OqFfea0dXdd9vqai=hGuQ8kuc9pgc9s8qqaq=dirpe0xb9q8qiLsFr0=vr0=vr0dc8meaabaqaciaacaGaaeqabaqabeGadaaakeaafaqadeGabaaabaGaeeyBa0MaeeyyaeMaeeiEaGNaeeyAaKMaeeyBa0MaeeyAaKMaeeOEaONaeeyzauMaeeiiaaIaeyOeI0YaaabCaeaadaaeWbqaaiabdohaZnaaBaaaleaacqWGPbqAcqWGQbGAaeqaaaqaaiabdQgaQjabg2da9iabigdaXaqaaiabd6gaUnaaBaaameaacqWG5bqEaeqaaaqdcqGHris5aaWcbaGaemyAaKMaeyypa0JaeGymaedabaGaemOBa42aaSbaaWqaaiabdIha4bqabaaaniabggHiLdGcdaqadiqaamaaemGabaGaemyqaeKaey4kaSIaemOuaiLaemiwaG1aaSbaaSqaaiabdMgaPbqabaGccqGHsislcqWGzbqwdaWgaaWcbaGaemOAaOgabeaaaOGaay5bSlaawIa7amaaCaaaleqabaGaeGOmaidaaOGaeyOeI0ccciGae83UdW2aaWbaaSqabeaacqaIYaGmaaaakiaawIcacaGLPaaaaeaafaqabeqacaaabaGaee4CamNaeeyDauNaeeOyaiMaeeOAaOMaeeyzauMaee4yamMaeeiDaqNaeeiiaaIaeeiDaqNaee4Ba8MaeeiiaaYaaeWaceaacqaIZaWmaiaawIcacaGLPaaacqGHsisldaqadiqaaiabisda0aGaayjkaiaawMcaaaqaaiabdohaZnaaBaaaleaacqWGPbqAcqWGQbGAaeqaaOGaeyicI48aaiWabeaacqaIWaamcqGGSaalcqaIXaqmaiaawUhacaGL9baaaaaaaiaaxMaacaWLjaWaaeWaceaacqaIXaqmcqaIWaamaiaawIcacaGLPaaaaaa@85B4@

which is an integer linear programming problem because of the binary variables *S*. But it can be exactly solved in polynomial time. As a matter of fact, (10) is a *maximum weighted bipartite matching problem *[17] which has the integrality property, i.e. the optimal solution is ensured to be integers even without the constraint *s*_*ij *_∈ {0,1}. Hence, the discrete optimal solution of LPS can be obtained by directly using any linear programming algorithm such as simplex algorithm or interior-point method by relaxing the binary variables as continuous variables 0 ≤ *s*_*ij *_≤ 1. However, there exists a more effective algorithm based on Hungarian method [17] to solve the maximum weighted bipartite matching problem. It is easy to show that the computational complexity of LPS with such a method is **O**(n¯
 MathType@MTEF@5@5@+=feaafiart1ev1aaatCvAUfKttLearuWrP9MDH5MBPbIqV92AaeXatLxBI9gBaebbnrfifHhDYfgasaacH8akY=wiFfYdH8Gipec8Eeeu0xXdbba9frFj0=OqFfea0dXdd9vqai=hGuQ8kuc9pgc9s8qqaq=dirpe0xb9q8qiLsFr0=vr0=vr0dc8meaabaqaciaacaGaaeqabaqabeGadaaakeaacuWGUbGBgaqeaaaa@2E29@(n^
 MathType@MTEF@5@5@+=feaafiart1ev1aaatCvAUfKttLearuWrP9MDH5MBPbIqV92AaeXatLxBI9gBaebbnrfifHhDYfgasaacH8akY=wiFfYdH8Gipec8Eeeu0xXdbba9frFj0=OqFfea0dXdd9vqai=hGuQ8kuc9pgc9s8qqaq=dirpe0xb9q8qiLsFr0=vr0=vr0dc8meaabaqaciaacaGaaeqabaqabeGadaaakeaacuWGUbGBgaqcaaaa@2E21@ + n¯
 MathType@MTEF@5@5@+=feaafiart1ev1aaatCvAUfKttLearuWrP9MDH5MBPbIqV92AaeXatLxBI9gBaebbnrfifHhDYfgasaacH8akY=wiFfYdH8Gipec8Eeeu0xXdbba9frFj0=OqFfea0dXdd9vqai=hGuQ8kuc9pgc9s8qqaq=dirpe0xb9q8qiLsFr0=vr0=vr0dc8meaabaqaciaacaGaaeqabaqabeGadaaakeaacuWGUbGBgaqeaaaa@2E29@logn¯
 MathType@MTEF@5@5@+=feaafiart1ev1aaatCvAUfKttLearuWrP9MDH5MBPbIqV92AaeXatLxBI9gBaebbnrfifHhDYfgasaacH8akY=wiFfYdH8Gipec8Eeeu0xXdbba9frFj0=OqFfea0dXdd9vqai=hGuQ8kuc9pgc9s8qqaq=dirpe0xb9q8qiLsFr0=vr0=vr0dc8meaabaqaciaacaGaaeqabaqabeGadaaakeaacuWGUbGBgaqeaaaa@2E29@)) where n¯
 MathType@MTEF@5@5@+=feaafiart1ev1aaatCvAUfKttLearuWrP9MDH5MBPbIqV92AaeXatLxBI9gBaebbnrfifHhDYfgasaacH8akY=wiFfYdH8Gipec8Eeeu0xXdbba9frFj0=OqFfea0dXdd9vqai=hGuQ8kuc9pgc9s8qqaq=dirpe0xb9q8qiLsFr0=vr0=vr0dc8meaabaqaciaacaGaaeqabaqabeGadaaakeaacuWGUbGBgaqeaaaa@2E29@ = *n*_*x *_+ *n*_*y *_and n^
 MathType@MTEF@5@5@+=feaafiart1ev1aaatCvAUfKttLearuWrP9MDH5MBPbIqV92AaeXatLxBI9gBaebbnrfifHhDYfgasaacH8akY=wiFfYdH8Gipec8Eeeu0xXdbba9frFj0=OqFfea0dXdd9vqai=hGuQ8kuc9pgc9s8qqaq=dirpe0xb9q8qiLsFr0=vr0=vr0dc8meaabaqaciaacaGaaeqabaqabeGadaaakeaacuWGUbGBgaqcaaaa@2E21@ = *n*_*x *_× *n*_*y*_.

Hungarian method is an efficient algorithm, but for large-scale problems, such as proteins with several hundreds amino acids, **O**(n¯
 MathType@MTEF@5@5@+=feaafiart1ev1aaatCvAUfKttLearuWrP9MDH5MBPbIqV92AaeXatLxBI9gBaebbnrfifHhDYfgasaacH8akY=wiFfYdH8Gipec8Eeeu0xXdbba9frFj0=OqFfea0dXdd9vqai=hGuQ8kuc9pgc9s8qqaq=dirpe0xb9q8qiLsFr0=vr0=vr0dc8meaabaqaciaacaGaaeqabaqabeGadaaakeaacuWGUbGBgaqeaaaa@2E29@(n^
 MathType@MTEF@5@5@+=feaafiart1ev1aaatCvAUfKttLearuWrP9MDH5MBPbIqV92AaeXatLxBI9gBaebbnrfifHhDYfgasaacH8akY=wiFfYdH8Gipec8Eeeu0xXdbba9frFj0=OqFfea0dXdd9vqai=hGuQ8kuc9pgc9s8qqaq=dirpe0xb9q8qiLsFr0=vr0=vr0dc8meaabaqaciaacaGaaeqabaqabeGadaaakeaacuWGUbGBgaqcaaaa@2E21@ + n¯
 MathType@MTEF@5@5@+=feaafiart1ev1aaatCvAUfKttLearuWrP9MDH5MBPbIqV92AaeXatLxBI9gBaebbnrfifHhDYfgasaacH8akY=wiFfYdH8Gipec8Eeeu0xXdbba9frFj0=OqFfea0dXdd9vqai=hGuQ8kuc9pgc9s8qqaq=dirpe0xb9q8qiLsFr0=vr0=vr0dc8meaabaqaciaacaGaaeqabaqabeGadaaakeaacuWGUbGBgaqeaaaa@2E29@logn¯
 MathType@MTEF@5@5@+=feaafiart1ev1aaatCvAUfKttLearuWrP9MDH5MBPbIqV92AaeXatLxBI9gBaebbnrfifHhDYfgasaacH8akY=wiFfYdH8Gipec8Eeeu0xXdbba9frFj0=OqFfea0dXdd9vqai=hGuQ8kuc9pgc9s8qqaq=dirpe0xb9q8qiLsFr0=vr0=vr0dc8meaabaqaciaacaGaaeqabaqabeGadaaakeaacuWGUbGBgaqeaaaa@2E29@)) is still too high for fast structure alignment. The algorithm for LPS can be further improved by exploiting its special feature. Notice that the objective function of (10) is to maximize−∑i=1nx∑j=1nysij(dij2−λ2)
 MathType@MTEF@5@5@+=feaafiart1ev1aaatCvAUfKttLearuWrP9MDH5MBPbIqV92AaeXatLxBI9gBaebbnrfifHhDYfgasaacH8akY=wiFfYdH8Gipec8Eeeu0xXdbba9frFj0=OqFfea0dXdd9vqai=hGuQ8kuc9pgc9s8qqaq=dirpe0xb9q8qiLsFr0=vr0=vr0dc8meaabaqaciaacaGaaeqabaqabeGadaaakeaacqqGTbqBcqqGHbqycqqG4baEcqqGPbqAcqqGTbqBcqqGPbqAcqqG6bGEcqqGLbqzcqGHsisldaaeWaqaamaaqadabaGaem4Cam3aaSbaaSqaaiabdMgaPjabdQgaQbqabaaabaGaemOAaOMaeyypa0JaeGymaedabaGaemOBa42aaSbaaWqaaiabdMha5bqabaaaniabggHiLdaaleaacqWGPbqAcqGH9aqpcqaIXaqmaeaacqWGUbGBdaWgaaadbaGaemiEaGhabeaaa0GaeyyeIuoakmaabmGabaGaemizaq2aa0baaSqaaiabdMgaPjabdQgaQbqaaiabikdaYaaakiabgkHiTiabeU7aSnaaCaaaleqabaGaeGOmaidaaaGccaGLOaGaayzkaaaaaa@5832@ for the fixed (*d*_*ij*_, *λ*). Therefore for *i *= 1, ..., *n*_*x *_and *j *= 1, ..., *n*_*y*_, if *d*_*ij *_≥ *λ*, then *s*_*ij *_= 0 must hold at the optimal solution. In other words, *λ *corresponds to the radius of the search region in the optimization process, and we can eliminate all *s*_*ij *_corresponding to *d*_*ij *_≥ *λ *from both the objective function and the constraints of (10). We can show that such a manipulation significantly simplifies LPS, and reduces total variables n^
 MathType@MTEF@5@5@+=feaafiart1ev1aaatCvAUfKttLearuWrP9MDH5MBPbIqV92AaeXatLxBI9gBaebbnrfifHhDYfgasaacH8akY=wiFfYdH8Gipec8Eeeu0xXdbba9frFj0=OqFfea0dXdd9vqai=hGuQ8kuc9pgc9s8qqaq=dirpe0xb9q8qiLsFr0=vr0=vr0dc8meaabaqaciaacaGaaeqabaqabeGadaaakeaacuWGUbGBgaqcaaaa@2E21@ from *n*_*x *_× *n*_*y *_to |{*d*_*ij *_: *d*_*ij *_<*λ*}| = **O**(*λ*^2^min{*n*_*x*_, *n*_*y*_}). An procedure for solving LPS based on Hungarian method with the reduced variables can be found in [15], and the algorithm ensures an integer solution without any approximation.

### Computational procedure for SAMO

Basically, (8) is optimized by solving LSS and LPS iteratively. In such a spirit, the algorithm of SAMO is summarized straightforward for a given *λ*.

• **Step-0**: Setting initial conditions:

- Assuming *n*_*x *_≤ *n*_*y*_, fix the coordinates of *Y*, and move *X *to their common center of mass by translation ∑i=1nyYi/ny−∑i=1nxXi/nx
 MathType@MTEF@5@5@+=feaafiart1ev1aaatCvAUfKttLearuWrP9MDH5MBPbIqV92AaeXatLxBI9gBaebbnrfifHhDYfgasaacH8akY=wiFfYdH8Gipec8Eeeu0xXdbba9frFj0=OqFfea0dXdd9vqai=hGuQ8kuc9pgc9s8qqaq=dirpe0xb9q8qiLsFr0=vr0=vr0dc8meaabaqaciaacaGaaeqabaqabeGadaaakeaadaaeWaqaaiabdMfaznaaBaaaleaacqWGPbqAaeqaaOGaei4la8IaemOBa42aaSbaaSqaaiabdMha5bqabaGccqGHsisldaaeWaqaaiabdIfaynaaBaaaleaacqWGPbqAaeqaaaqaaiabdMgaPjabg2da9iabigdaXaqaaiabd6gaUnaaBaaameaacqWG4baEaeqaaaqdcqGHris5aaWcbaGaemyAaKMaeyypa0JaeGymaedabaGaemOBa42aaSbaaWqaaiabdMha5bqabaaaniabggHiLdGccqGGVaWlcqWGUbGBdaWgaaWcbaGaemiEaGhabeaaaaa@4BCE@. Set *λ*, annealing coefficients *γ *and λ¯
 MathType@MTEF@5@5@+=feaafiart1ev1aaatCvAUfKttLearuWrP9MDH5MBPbIqV92AaeXatLxBI9gBaebbnrfifHhDYfgasaacH8akY=wiFfYdH8Gipec8Eeeu0xXdbba9frFj0=OqFfea0dXdd9vqai=hGuQ8kuc9pgc9s8qqaq=dirpe0xb9q8qiLsFr0=vr0=vr0dc8meaabaqaciaacaGaaeqabaqabeGadaaakeaaiiGacuWF7oaBgaqeaaaa@2E7F@, and convergence criterion *ε*, which are all positive numbers. Set all initial values of variables *s*_*ij*_, and let the iteration index *t *= 1.

• **Step-1**: Solving LSS:

- Solve (9) for (*A*, *R*) with the fixed *S *by the SVD algorithm (see [7]).

• **Step-2**: Solving LPS:

- Solve (10) for *S *with the given (*A*, *R*) in Step-1 by the procedure based on Hungarian method. Reduce variables based on *λ *as explained in Section LPS.

• **Step-3**: Checking convergence:

- When |*T*^(*t*) ^- *T*^(*t*-1)^| ≤ *ε *is satisfied, terminate the computation and output *rms *and *m*. Otherwise, let *t *← *t *+ 1.

• **Step-4**: Annealing process:

- Let *λ*^(*t*) ^= *λ *+ λ¯
 MathType@MTEF@5@5@+=feaafiart1ev1aaatCvAUfKttLearuWrP9MDH5MBPbIqV92AaeXatLxBI9gBaebbnrfifHhDYfgasaacH8akY=wiFfYdH8Gipec8Eeeu0xXdbba9frFj0=OqFfea0dXdd9vqai=hGuQ8kuc9pgc9s8qqaq=dirpe0xb9q8qiLsFr0=vr0=vr0dc8meaabaqaciaacaGaaeqabaqabeGadaaakeaaiiGacuWF7oaBgaqeaaaa@2E7F@*γ*^*t *^where *λ *is the target value, λ¯
 MathType@MTEF@5@5@+=feaafiart1ev1aaatCvAUfKttLearuWrP9MDH5MBPbIqV92AaeXatLxBI9gBaebbnrfifHhDYfgasaacH8akY=wiFfYdH8Gipec8Eeeu0xXdbba9frFj0=OqFfea0dXdd9vqai=hGuQ8kuc9pgc9s8qqaq=dirpe0xb9q8qiLsFr0=vr0=vr0dc8meaabaqaciaacaGaaeqabaqabeGadaaakeaaiiGacuWF7oaBgaqeaaaa@2E7F@ ≥ 0 and 1 > *γ *> 0 (a cooling coefficient for annealing). Then go to Step-1 with the updated *S*.

For Step-0, the original centers of mass for proteins *X *and *Y *are X¯=∑i=1nxXi/nx
 MathType@MTEF@5@5@+=feaafiart1ev1aaatCvAUfKttLearuWrP9MDH5MBPbIqV92AaeXatLxBI9gBaebbnrfifHhDYfgasaacH8akY=wiFfYdH8Gipec8Eeeu0xXdbba9frFj0=OqFfea0dXdd9vqai=hGuQ8kuc9pgc9s8qqaq=dirpe0xb9q8qiLsFr0=vr0=vr0dc8meaabaqaciaacaGaaeqabaqabeGadaaakeaacuWGybawgaqeaiabg2da9maaqadabaGaemiwaG1aaSbaaSqaaiabdMgaPbqabaaabaGaemyAaKMaeyypa0JaeGymaedabaGaemOBa42aaSbaaWqaaiabdIha4bqabaaaniabggHiLdGccqGGVaWlcqWGUbGBdaWgaaWcbaGaemiEaGhabeaaaaa@3E10@ and Y¯=∑i=1nyYi/ny
 MathType@MTEF@5@5@+=feaafiart1ev1aaatCvAUfKttLearuWrP9MDH5MBPbIqV92AaeXatLxBI9gBaebbnrfifHhDYfgasaacH8akY=wiFfYdH8Gipec8Eeeu0xXdbba9frFj0=OqFfea0dXdd9vqai=hGuQ8kuc9pgc9s8qqaq=dirpe0xb9q8qiLsFr0=vr0=vr0dc8meaabaqaciaacaGaaeqabaqabeGadaaakeaacuWGzbqwgaqeaiabg2da9maaqadabaGaemywaK1aaSbaaSqaaiabdMgaPbqabaaabaGaemyAaKMaeyypa0JaeGymaedabaGaemOBa42aaSbaaWqaaiabdMha5bqabaaaniabggHiLdGccqGGVaWlcqWGUbGBdaWgaaWcbaGaemyEaKhabeaaaaa@3E18@ respectively. *rms *or RMSD is defined as T/m
 MathType@MTEF@5@5@+=feaafiart1ev1aaatCvAUfKttLearuWrP9MDH5MBPbIqV92AaeXatLxBI9gBaebbnrfifHhDYfgasaacH8akY=wiFfYdH8Gipec8Eeeu0xXdbba9frFj0=OqFfea0dXdd9vqai=hGuQ8kuc9pgc9s8qqaq=dirpe0xb9q8qiLsFr0=vr0=vr0dc8meaabaqaciaacaGaaeqabaqabeGadaaakeaadaGcaaqaaiabdsfaujabc+caViabd2gaTbWcbeaaaaa@3041@, where *T *and *m *are expressed in (5)–(6). Step-4 of Algorithm is for the annealing. That is, first a large initial *λ*^(0) ^= *λ *+ λ¯
 MathType@MTEF@5@5@+=feaafiart1ev1aaatCvAUfKttLearuWrP9MDH5MBPbIqV92AaeXatLxBI9gBaebbnrfifHhDYfgasaacH8akY=wiFfYdH8Gipec8Eeeu0xXdbba9frFj0=OqFfea0dXdd9vqai=hGuQ8kuc9pgc9s8qqaq=dirpe0xb9q8qiLsFr0=vr0=vr0dc8meaabaqaciaacaGaaeqabaqabeGadaaakeaaiiGacuWF7oaBgaqeaaaa@2E7F@ is set so that the algorithm performs a global search on a large region to find a better matching in the earlier iterations. Then, reduce *λ*^(*t*) ^= *λ *+ λ¯
 MathType@MTEF@5@5@+=feaafiart1ev1aaatCvAUfKttLearuWrP9MDH5MBPbIqV92AaeXatLxBI9gBaebbnrfifHhDYfgasaacH8akY=wiFfYdH8Gipec8Eeeu0xXdbba9frFj0=OqFfea0dXdd9vqai=hGuQ8kuc9pgc9s8qqaq=dirpe0xb9q8qiLsFr0=vr0=vr0dc8meaabaqaciaacaGaaeqabaqabeGadaaakeaaiiGacuWF7oaBgaqeaaaa@2E7F@*γ*^*t *^by *γ *to narrow the searching region during each iteration until convergence. Although introducing the annealing process requires additional computation cost, it enlarges the searching region which results in the improvement of alignment quality. Such an annealing process is only activated when the quality of the alignment is not satisfied. For the computation without annealing, simply set λ¯
 MathType@MTEF@5@5@+=feaafiart1ev1aaatCvAUfKttLearuWrP9MDH5MBPbIqV92AaeXatLxBI9gBaebbnrfifHhDYfgasaacH8akY=wiFfYdH8Gipec8Eeeu0xXdbba9frFj0=OqFfea0dXdd9vqai=hGuQ8kuc9pgc9s8qqaq=dirpe0xb9q8qiLsFr0=vr0=vr0dc8meaabaqaciaacaGaaeqabaqabeGadaaakeaaiiGacuWF7oaBgaqeaaaa@2E7F@ = 0.

### Parameter selection

As discussed in previous sections, *s*_*ij *_is possibly 1 at the optimal solution only if *d*_*ij *_<*λ*. If the distance of any two atoms *i *and *j *is bigger than *λ*, no matching for such two atoms is considered in LPS. In other words, only atom pairs with the distance less than *λ *are further considered in LSS for the translation and rotation operation because of *s*_*ij *_= 0 for any atom pairs with *d*_*ij *_> *λ*. As a result, the aligned *rms *in LSS is less than *λ*. We can use this property to obtain an alignment for a specific *rms *by setting an appropriate *λ *but without searching the Pareto set completely. Empirically, we can obtain an optimal solution with *rms *= *r *if setting *λ *= 2*r *~ 3*r*, where *rms *is expected to be *r *= 0 ~ 3 because an alignment for *rms *> 3 is not generally considered as a good matching. In other words, we can give a list of solutions that covers all the reasonable alignments with *λ *changing from 0 to 9 because the range of *rms *for those solutions generally in [0, 3]. In the implement of the software SAMO, we set the default value of the parameter *λ *as 6.0 according to this rule. Actually, in most cases it always gives satisfactory alignment results. Considering that the distance is approximately 3.8Å for two consecutive *C*_*α *_atoms or *C*_*β *_atoms in a protein chain, the reduced LPS generally has variables less than min{*n*_*x*_, *n*_*y*_}(*λ *+ 3.8)^2^/3.8^2^, which is a much smaller Linear Programming (LP) than the original LP with variables *n*_*x *_× *n*_*y*_. For example, the number of variables is approximately less than 400 × (6 + 3.8)^2^/3.8^2 ^≈ 2660 for a pair of proteins both with 400 amino acids and *λ *= 6, while there are 400 × 400 = 160000 variables in the original LP.

### Convergence analysis

The decomposition of the algorithm actually ensures the local convergence. We next prove the convergence of the proposed algorithm. Let *A*^*k *^and *R*^*k *^be the solution of LSS (9) at the *k*-th iteration with an assignment *S*^*k*-1^. Then

∑i=1nx∑j=1nysijk−1(|Ak+RkXi−Yj|2−λ2)≤∑i=1nx∑j=1nysijk−1(|Ak−1+Rk−1Xi−Yj|2−λ2)
 MathType@MTEF@5@5@+=feaafiart1ev1aaatCvAUfKttLearuWrP9MDH5MBPbIqV92AaeXatLxBI9gBaebbnrfifHhDYfgasaacH8akY=wiFfYdH8Gipec8Eeeu0xXdbba9frFj0=OqFfea0dXdd9vqai=hGuQ8kuc9pgc9s8qqaq=dirpe0xb9q8qiLsFr0=vr0=vr0dc8meaabaqaciaacaGaaeqabaqabeGadaaakeaadaaeWbqaamaaqahabaGaem4Cam3aa0baaSqaaiabdMgaPjabdQgaQbqaaiabdUgaRjabgkHiTiabigdaXaaaaeaacqWGQbGAcqGH9aqpcqaIXaqmaeaacqWGUbGBdaWgaaadbaGaemyEaKhabeaaa0GaeyyeIuoaaSqaaiabdMgaPjabg2da9iabigdaXaqaaiabd6gaUnaaBaaameaacqWG4baEaeqaaaqdcqGHris5aOWaaeWaceaadaabdiqaaiabdgeabnaaCaaaleqabaGaem4AaSgaaOGaey4kaSIaemOuai1aaWbaaSqabeaacqWGRbWAaaGccqWGybawdaWgaaWcbaGaemyAaKgabeaakiabgkHiTiabdMfaznaaBaaaleaacqWGQbGAaeqaaaGccaGLhWUaayjcSdWaaWbaaSqabeaacqaIYaGmaaGccqGHsisliiGacqWF7oaBdaahaaWcbeqaaiabikdaYaaaaOGaayjkaiaawMcaaiabgsMiJoaaqahabaWaaabCaeaacqWGZbWCdaqhaaWcbaGaemyAaKMaemOAaOgabaGaem4AaSMaeyOeI0IaeGymaedaaaqaaiabdQgaQjabg2da9iabigdaXaqaaiabd6gaUnaaBaaameaacqWG5bqEaeqaaaqdcqGHris5aaWcbaGaemyAaKMaeyypa0JaeGymaedabaGaemOBa42aaSbaaWqaaiabdIha4bqabaaaniabggHiLdGcdaqadiqaamaaemGabaGaemyqae0aaWbaaSqabeaacqWGRbWAcqGHsislcqaIXaqmaaGccqGHRaWkcqWGsbGudaahaaWcbeqaaiabdUgaRjabgkHiTiabigdaXaaakiabdIfaynaaBaaaleaacqWGPbqAaeqaaOGaeyOeI0IaemywaK1aaSbaaSqaaiabdQgaQbqabaaakiaawEa7caGLiWoadaahaaWcbeqaaiabikdaYaaakiabgkHiTiab=T7aSnaaCaaaleqabaGaeGOmaidaaaGccaGLOaGaayzkaaaaaa@9095@

Using *A*^*k *^and *R*^*k*^, we solve LPS (10), and let the solution be *S*^*k*^. Then there is

∑i=1nx∑j=1nysijk(|Ak+RkXi−Yj|2−λ2)≤∑i=1nx∑j=1nysijk−1(|Ak+RkXi−Yj|2−λ2)
 MathType@MTEF@5@5@+=feaafiart1ev1aaatCvAUfKttLearuWrP9MDH5MBPbIqV92AaeXatLxBI9gBaebbnrfifHhDYfgasaacH8akY=wiFfYdH8Gipec8Eeeu0xXdbba9frFj0=OqFfea0dXdd9vqai=hGuQ8kuc9pgc9s8qqaq=dirpe0xb9q8qiLsFr0=vr0=vr0dc8meaabaqaciaacaGaaeqabaqabeGadaaakeaadaaeWbqaamaaqahabaGaem4Cam3aa0baaSqaaiabdMgaPjabdQgaQbqaaiabdUgaRbaaaeaacqWGQbGAcqGH9aqpcqaIXaqmaeaacqWGUbGBdaWgaaadbaGaemyEaKhabeaaa0GaeyyeIuoaaSqaaiabdMgaPjabg2da9iabigdaXaqaaiabd6gaUnaaBaaameaacqWG4baEaeqaaaqdcqGHris5aOWaaeWaceaadaabdiqaaiabdgeabnaaCaaaleqabaGaem4AaSgaaOGaey4kaSIaemOuai1aaWbaaSqabeaacqWGRbWAaaGccqWGybawdaWgaaWcbaGaemyAaKgabeaakiabgkHiTiabdMfaznaaBaaaleaacqWGQbGAaeqaaaGccaGLhWUaayjcSdWaaWbaaSqabeaacqaIYaGmaaGccqGHsisliiGacqWF7oaBdaahaaWcbeqaaiabikdaYaaaaOGaayjkaiaawMcaaiabgsMiJoaaqahabaWaaabCaeaacqWGZbWCdaqhaaWcbaGaemyAaKMaemOAaOgabaGaem4AaSMaeyOeI0IaeGymaedaaaqaaiabdQgaQjabg2da9iabigdaXaqaaiabd6gaUnaaBaaameaacqWG5bqEaeqaaaqdcqGHris5aaWcbaGaemyAaKMaeyypa0JaeGymaedabaGaemOBa42aaSbaaWqaaiabdIha4bqabaaaniabggHiLdGcdaqadiqaamaaemGabaGaemyqae0aaWbaaSqabeaacqWGRbWAaaGccqGHRaWkcqWGsbGudaahaaWcbeqaaiabdUgaRbaakiabdIfaynaaBaaaleaacqWGPbqAaeqaaOGaeyOeI0IaemywaK1aaSbaaSqaaiabdQgaQbqabaaakiaawEa7caGLiWoadaahaaWcbeqaaiabikdaYaaakiabgkHiTiab=T7aSnaaCaaaleqabaGaeGOmaidaaaGccaGLOaGaayzkaaaaaa@8AFE@

which shows that the value of the objective function *T*(*S*^*k*^, *A*^*k*^, *R*^*k*^) always decreases with the iteration of the computation. Noticing that the objective function has a special structure and the solution space of *S*^*k *^is a finite set, decrease of the objective function implies that *A*^*k *^+ *R*^*k*^*X*_*i *_will be in a bounded neighborhood of the point *Y*_*i*_. Therefore, there will be a subsequence of the solution sequence that converges to a cluster so that the termination condition will be satisfied to end the computation.

Note that although our algorithm can obtain an optimal alignment for any specified *λ*, the resulting solution may not be globally optimal because of the non-convexity of the protein structure alignment problem. In other words, depending on initial condition, the algorithm may result in a different solution, as the same as most of deterministic optimization techniques do. To improve the quality of the solution, we can further adopt annealing technique to enlarge the searching space [15]. Specifically, in our program we set λ¯
 MathType@MTEF@5@5@+=feaafiart1ev1aaatCvAUfKttLearuWrP9MDH5MBPbIqV92AaeXatLxBI9gBaebbnrfifHhDYfgasaacH8akY=wiFfYdH8Gipec8Eeeu0xXdbba9frFj0=OqFfea0dXdd9vqai=hGuQ8kuc9pgc9s8qqaq=dirpe0xb9q8qiLsFr0=vr0=vr0dc8meaabaqaciaacaGaaeqabaqabeGadaaakeaaiiGacuWF7oaBgaqeaaaa@2E7F@ = 10*λ*, and *γ *= 0.4 the annealing procedure when the rms is undesirably large. In other words, the radius of initial searching region with the annealing is enlarged by 11 times.

Numerical simulations indicate that alignment of a protein pair typically requires 4–10 iterations, and the convergence is always achieved from the numerical computation viewpoint.

**Figure 2:**
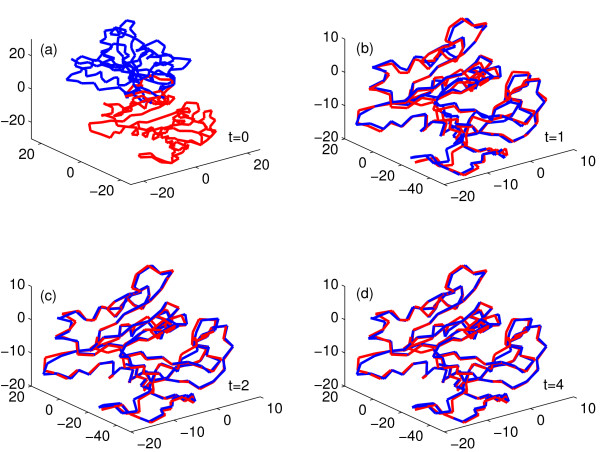
An example of the alignment for two protein chains 1*DHF*_*a *_and 8*DFR*. The iteration number of the algorithm is represented by *t*. (a) The two proteins 1*DHF*_*a *_and 8*DFR *are in the original coordinates, which are in the *C*_*α *_representation of the backbone. (b) and (c) Relative positions of the two proteins during the convergence process. (d) The optimal alignment by our algorithm. The final alignment number *m *is 182 with *rms *= 0.7 at *λ *= 6.0 and λ¯
 MathType@MTEF@5@5@+=feaafiart1ev1aaatCvAUfKttLearuWrP9MDH5MBPbIqV92AaeXatLxBI9gBaebbnrfifHhDYfgasaacH8akY=wiFfYdH8Gipec8Eeeu0xXdbba9frFj0=OqFfea0dXdd9vqai=hGuQ8kuc9pgc9s8qqaq=dirpe0xb9q8qiLsFr0=vr0=vr0dc8meaabaqaciaacaGaaeqabaqabeGadaaakeaaiiGacuWF7oaBgaqeaaaa@2E7F@ = 0.0.

Figure 2 is a typical example of the alignment process for a pair of proteins 1*DHFa *and 8*DFR *with translation, rotation and matching of atoms. As shown in (a)–(d) of Figure 2, the protein 1*DHF*_*a *_rotates and translates by approaching to 8*DFR *very fast with only four iterations, (d) is the converged result, and λ¯
 MathType@MTEF@5@5@+=feaafiart1ev1aaatCvAUfKttLearuWrP9MDH5MBPbIqV92AaeXatLxBI9gBaebbnrfifHhDYfgasaacH8akY=wiFfYdH8Gipec8Eeeu0xXdbba9frFj0=OqFfea0dXdd9vqai=hGuQ8kuc9pgc9s8qqaq=dirpe0xb9q8qiLsFr0=vr0=vr0dc8meaabaqaciaacaGaaeqabaqabeGadaaakeaaiiGacuWF7oaBgaqeaaaa@2E7F@ = 0 means that there is no annealing process in the computation. Note that the coordinate of 8*DFR *is fixed due to its longer amino sequence according to the algorithm.

## Results

The algorithm was implemented in C++ language. The simulation for each structure alignment of a pair of proteins generally requires a few minutes (most of them are less than 10 seconds) on Pentium 4 CPU, which is considered fast. For example, alignment of proteins 1*DHFa *and 8*DFR *which have lengths 182 and 186 respectively can find a long segment with length 182 and RMSD 0.72 in only 6 seconds. The alignment results can be presented in various output styles and saved for further analysis. The detailed residue correspondence is provided and can be saved in PDB file format for the purpose of the visualization. The software SAMO [see Additional file 1,2,3] is available upon request from authors or from [18]. We have conducted the comparison experiments using dozens proteins (benchmark examples) from major protein families and folds, as shown in Table 1. The comparisons are carried out both in same family, fold, and in different family, fold. These results of comparison can be roughly summarized as three categories according to their comparison scores.

The first category is composed of the protein pairs with lower RMSD value and larger number of matched amino-acid pairs, which conserve the sequential orders (even though no sequential order constraints are used). The result of the proposed method indicates global similarity between two whole structures. Because the geometric match corresponds to a sequential order, protein pairs in this category may imply evolutionary divergence. In the section "Revealing Divergent Evolution", we will compare our method (SAMO) with the conventional methods and further clarify the results.

In this paper, we analyze the convergent and divergent evolutions by structure comparisons. Divergent evolution is the process of two or more related species becoming more and more dissimilar from a common ancestor. Similarities in sequence and structure indicate that the two species have a common ancestor. As they adapted to different environments, the structures of the two species diverged. In convergent evolution, on the other hand, unrelated species from different ancestors become more and more similar in appearance or structure (not necessarily in sequence) as they possibly adapt to the same kind of environment. Convergent evolution takes place when species of different ancestry begin to share analogous traits because of a shared environment or other selection pressure. Although it is generally difficult to distinguish such two evolutions, our method in this paper can provide some insight about this problem.

The second category is composed of the protein pairs with higher RMSD value and smaller number of matched amino-acid pairs. The result of the proposed method mainly demonstrates local similarity between substructures. These matches contain fewer matched amino-acid pairs than the first category, and do not necessarily conserve the sequential order. Proteins with such substructural similarity may imply evolutionary convergence. At first sight, it seems that it is a random match of small segments plus isolated residues. However close inspection reveals that many of the matched amino-acid pairs perform some common biological functions. Since our comparison is conducted in a structural level without any sequential order constraint, the match is completely 3D. We define these recurring detected substructures as structure motifs or active-sites in this paper. They are "real" 3D motifs, which are different from the conventional concept of motifs defined by the multiple sequence alignment. In the section "Identifying Active Sites", we will report the ability of our method (SAMO) to find similarity of active sites or 3D structural motifs. The third category corresponds to spurious matches between unrelated and dissimilar structures. These may contain equivalences of single *α*-helix, *β*-strands, or randomly matched isolated residues. The protein pairs in this category have higher RMSD and smaller number of matched amino-acid pairs, which are not located in a local area. Those pairs can be regarded as the negative samples identified by our method (SAMO), which means that the two proteins are not similar biologically, and can be excluded from the results by checking biologically meaningful similarity of the matched residues.

Circular permutation is a special phenomenon in structure database formed by mutation in the sequential order. It provides challenge for the structure alignment methods with sequential order constraint. We show the advantage of our method (SAMO) in finding circular permutation due to the sequence order-independent strategy in the section "Detecting Circular Permutations". In addition, other features of our method (SAMO) are also reported in the section "Results", such as stable convergence and high computation efficiency.

### Revealing divergent evolution

In this paper, we compare the 3D protein structures in the multi-objective optimization framework without the sequential order constraint. This allows us to detect similarity between protein molecules, and find out whether those amino acids are on the surfaces or in the interior. This truly 3D comparison approach overcomes a limitation inherent in other conventional structure alignment techniques which require that the linear order of the amino acid sequences be conserved. In this section, we will compare SAMO with the conventional methods, such as Dali, CE and Lund.

First we emphasize that one of the basic roles of protein comparison is to provide insight into evolution. i.e. address the question of divergence or convergence of proteins [14]. Originally, interest in automated structural comparison methods arose from the need to superimpose the structures of divergently evolved proteins. In such comparisons, a strict sequential order conservation has been enforced. In this paper, we show that both SAMO and the conventional methods perform well for the comparison of divergently evolved proteins (in the first category).

However, SAMO can deduce additional evidence of divergent evolution when the results of a pure 3D structural comparison reveal that sequential order is conserved. In other words, SAMO "rediscovers" the dual sequence-structure homology in divergent species. We will clarify this point in the following examples. We adopt the same benchmark examples as those of [10,7,6] from Protein Data Bank [19] as a basic set for numerical simulation by comparing with the several existing methods, i.e. Dali [9,20], CE [10,21], and Lund [6]. There is no post processing in our simulation, and *C*_*α *_representation is adopted for each protein chain. The convergence criterion is *ε *= 0.01 for all examples.

The simulation results are shown in Table 1, where Dihydrofolate reductases and Globins are considered easy for alignment while other ten protein pairs are thought to be very difficult to align [10]. For any protein pair, SAMO gives a list of solutions corresponding to different *λ *from small value to large value, which all belong to the Pareto set. Since a different *λ *gives an optimal solution with different *m *and *rms *for the proposed method, we listed those results with the corresponding *λ*, which are comparable to others. According to Table 1, all of the aligned results by SAMO are almost consistently better than others. The comparison results in Reductases and Globins family show that both SAMO and the conventional methods can obtain good matches. All of the *rms *are lower than 2.5 and almost all the residues are matched. The difference lies in that our results are obtained without the sequential order constraint. In particular, for the ten most difficult protein pairs [10], our algorithm performs effectively and typically produces alignments with much lower *rms *distances or longer chains. Since most of the matched amino-acid pairs by SAMO conserve sequential order, the protein pairs for "Reductases", "Globins" and "Ten difficult structures" of Table 1 belong to the first category, which indicates that those pairs are evolutionarily divergent, and are originally from the same family.

In addition, we also aligned protein pairs for different folds and different classes, and compared the results with other methods. The results indicated that our algorithm can obtain an alignment with a larger matching portion with a better RMSD for those protein pairs. "-" in the table means that method does not give a result or the result is not available. Simple analysis indicates that the conventional methods perform well for proteins belonging to the same family or fold, but it is difficult for them to detect similarities of proteins belonging to different folds and classes unless there are sufficiently large fragments of consecutive residues in both proteins. In contrast, our approach overcomes these limitations. In particular, in addition to aligning protein structures for the first category (typically with a lower *rms *and more aligned pairs), it is able to obtain matches of isolated residues not belonging to contiguous fragments or belonging to non-secondary structure elements, in particular, structure motifs. To demonstrate that the aligned 3D structure motif by SAMO has biological meaning, we give several examples of detecting similarity for active sites in subsection "Identifying Active Sites", which belong to the second category. Furthermore, in the last part of Table 1, two circular permutation examples are given. The results show that SAMO also outperforms other methods. At the same level of *rms*, the number of matched amino-acid pairs in SAMO experiments is almost double. More examples for finding circular permutation are discussed in details in subsection "Detecting Circular Permutations".

### Detecting circular permutations

A circularly permuted protein arises from protein duplication and subsequent deletion of N- and C-terminal regions in the corresponding duplicated units. The motivation of emphasis on comparing such proteins is partly originated in that circularly permuted proteins are common in the protein structure database. As reported in [22], there are 47% of all protein domains are superimposable to at least one other protein domain in the database after their sequences are circularly permuted by a systematic search for all protein pairs in the SCOP domain database. Especially some of them are nonsymmetric proteins, which become structurally superimposable to other protein only after a circular permutation of the sequence. In such a way, their remote homology can be detected. Also discovery of circular permutation at genome wide scale will enable systematic studies of its contribution to the generation of novel protein function and novel protein fold.

**Figure 3:**
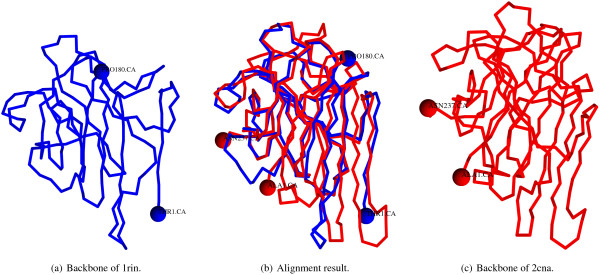
The comparison result of a naturally occurring protein pair 1RIN/180 and 2CNA/237 by SAMO. The subfigures (a) and (c) are backbone of proteins 1RIN and 2CNA respectively. The subfigure (c) illustrates the aligned result after the optimal superimposing by different colors. The red chain is 2CNA and the blue chain is 1RIN. The two termini of the two structures are indicated by labeling their residue names respectively. Notice that the termini of the blue chain (1RIN) are aligned to the middle of the red chain (2CNA) and vice versa.

Currently there are mainly two classes of the available circularly permuted detecting methods. One is sequence alignment-based methods [23]. Its drawback lies in that it can miss many circularly permuted proteins, because either one or both fragments may escape detection by local alignment if the two proteins are distantly related. The second class is structure alignment-based methods. As shown in Table 1, the conventional methods such as, Dali and CE fail to detect circular permutation due to sequential order constraint in computation. One feasible way is to use structure alignment in an order-independent manner [7,24], which is promising to uncover many more ancient permutation events. In this subsection, we will focus on detecting circularly permuted proteins by comparing with the method of [24] on a larger set of examples. These results are listed in Table 2. With the parameter A taking the default value 6.0, SAMO outperforms the method in [24] both in naturally occurring and human made examples of permuted proteins. The match between naturally occurring pair 1RIN and 2CNA is illustrated in Figure 3.

### Identifying active sites

Recognition of common substructural features (the pure structure motif) that do not generally conserve the amino-acid sequential order entails application of the sequence order-independent methods. Examples of such features may include similarities between active sites of convergent structures, between different folding motifs, between scaffolds of unrelated proteins, and between recurring stable configurations in the interior of proteins. In contrast to the concept of motifs defined by the multiple sequence alignment, we aim to identify structural motifs or active sites which are "real" 3D motifs. As shown in this section, SAMO succeeded in detecting the similarities of active sites or structural motifs.

**Figure 4:**
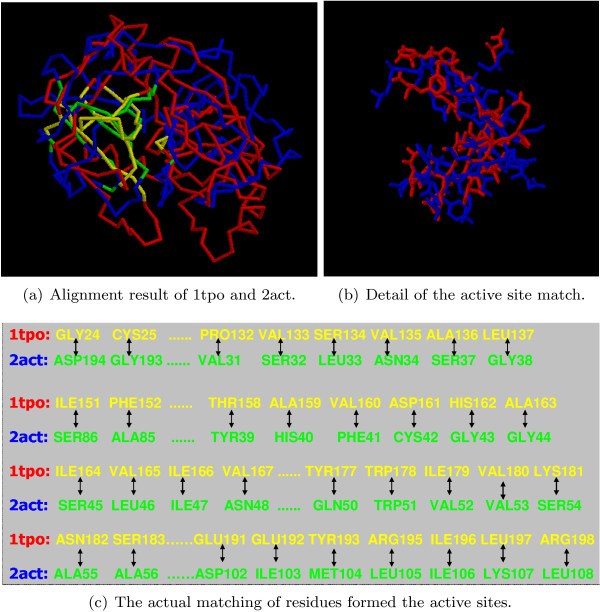
The comparison result of protein pair 1TPO/223 (*β*-trysin) and 2ACT/218 (actinidin) by SAMO. Subfigure (a) illustrates the aligned result after the optimal superimposing by different colors. The red chain is 1TPO and the blue chain is 2ACT. There are 122 amino-acid matching pairs with RMSD = 2.02. Also the active site region on 1TPO is highlighted in yellow and the active site region on 2act is drawn in green. Subfigure (b) is the detail match of the active sites between 1TPO(red) and 2ACT(blue). As indicated in the figure, the superposition of *C*_*α*_*s *found by SAMO brings the catalytic triads close together. In the matched active sites, the amino acids come from different fragments of each protein chain. Some segments contain contiguous residues. The actual matching of these segments is, for 2ACT: 24–25, 151–152, 132–137, 158–167, 177–183, 191–198; for 1TPO: 194–195, 85–86, 31–36, 39–48, 50–56, 101–108. The actual matching of the residues formed the active sites is listed in Subfigure (c).

The structural similarity between the active sites of proteins only can be recognized by visual inspection. Similar to the results in [14], SAMO succeeded in finding the rough similarity around the active sites of proteins automatically, without any prior knowledge of their existence and the information of side chains. For example in Figure 4, we give the comparison result of proteins *β*-trypsin (1TPO) and actinidin (2ACT). At first sight, it seems that it is a random match of small segments plus isolated residues (subfigure (a) of Figure 4, active sites are highlighted in different colors). However close inspection reveals that most of the matched pairs are located in the active sites. The subfigures (b) and (c) of Figure 4 show the detailed match of the detected active-sites by removing the aligned isolated residues which are not biological meaningful. Clearly the residues composing the active site come from different regions of the protein chain. The similarity is evaluated from pure structural viewpoint and can only be detected in a sequence order-independent strategy. Our findings are similar to the results in [14] (ref. Fig. 3) but have more matched amino-acid pairs. In addition, for another example (*β*-trypsin (1TPO) and proteinase K (2PRK)) in [14], we also obtain better results.

The comparison with the order-independent structure comparison method in [14] was conducted and the results are listed in Table 3. The protein pairs are taken from the Table 1 of [14], where some entries are removed due to the structure data update of PDB database. Another criterion (Score in Table 3) proposed in [14] is introduced to assess the quality of protein structure comparison. The Score is defined as: Score = *m*/((*n*_*x *_- *m*) + (*n*_*y *_- *m*) + *m*) = *m*/(*n*_*x *_+ *n*_*y *_- *m*), where *m *is the number of matched amino-acid pairs between two proteins; *n*_*x *_and *n*_*y *_are the number of amino acids of the two proteins *X *and *Y *respectively. Clearly, the number of matched amino-acid pairs is divided by the sum of the number of unmatched residues in protein *X*, the number of unmatched residues in protein *Y *and the number of matched amino-acid pairs. This score is designed to take into account the number of matched amino-acid pairs and to penalize the difference in sizes between two proteins. The comparison results show that SAMO performs better when considering all three criteria together: *m *(number of matched amino-acid pairs), *rms *(root mean square distance) and Score.

## Discussion

As demonstrated in the paper, SAMO has the following features in addition to structure alignment: 1) detect spatial similarity between evolutionary convergent or divergent structures; 2) identify active sites (structural motifs) and circular permutations; 3) reduce the computational complexity and improve the comparison quality.

For evolutionarily related proteins, the alignment results by SAMO show the strict sequential order. Hence, our method (SAMO) not only can detect this kind of structure similarity, but also can provide stronger evidence in favor of divergent evolution comparing with the conventional structure alignment methods. Also SAMO has the ability to find circular permutations by structure comparison.

By matching isolated residues, one of major benefits for SAMO is that it can find the similar three dimensional motifs (structural motifs) between proteins which belong to different families or different folds, although many of these motifs have not be generally found and their biological functions are not well identified. Another potential application is to use SAMO to detect similarity for pockets or mouths in protein surfaces that are closely related to protein functions [25]. In fact, one reason to develop the method is because the detection of similar protein surface patterns with different underlying primary sequence order can not be addressed by the current structure alignment method. The need to develop such a method is further illustrated in [25]. For example, when convergent evolution occurs, nature discovers the same functional surfaces multiple times, as is the case of the catalytic triad in serine protease. It is likely that there may be many such examples where proteins with similar functional surfaces have different underlying protein core architectures, and in particular, the key residues important for function may have different order in primary sequences. Our method currently can detect such similarity and can be used in assessing similarity of order-independent surface patterns. The comparison for protein pockets by SAMO and the results assessment are currently in progress.

Although the proposed algorithm can find the structural motifs by comparing protein pairs, the aligned residues may not always represent biologically meaningful substructures or regions. One reason is that the aligned atom pairs may be distributed in a wide area or may not be always restricted in a local area of a protein. To exclude such cases (in the third category), manual inspection is needed to find biologically meaningful match of residues.

As reported in this paper, another major contribution of the new method is its concise in mathematics and cheap in computation. We expect that our method will enable routine comparisons of any picked structure against the large database of 3D structures and provide web service by exploiting current information technology in the similar manner to the comparison of a query DNA sequence with the sequence database. It will further provide a wealth of information and an insight into evolutionary and functional aspects of biological macromolecules. The implication of the availability of such a tool can provide applications ranging from protein folding problem to computer-aided drug design because it is the structure that plays a critical role in carrying out the necessary biological functions. The software SAMO is available at the website [18].

## Conclusion

In this paper we developed SAMO which is able to align protein structures, reveal divergent evolution, detect circular permutations and identify structural motifs in an accurate and efficient manner. The proposed algorithm is general and treats the structure alignment in a more accurate way with implicit complete exploration of the entire space. The original protein alignment problem is formulated as a multi-objective optimization problem with mixed variables, and further decomposed into LPS and LSS. A very efficient algorithm with a numerically stable convergent process is developed for solving LPS and LSS successively. We show that the size of variables linearly increases with respect to the number of atoms of the protein pairs. By controlling a single distance-related parameter, theoretically we can obtain a variety of optimal alignments corresponding to different optimal matching patterns, i.e., from a large matching portion to a small matching portion. Numerical results further support that SAMO can not only detect close spatial similarity between evolutionarily divergent structures and circular permutations but also identify remote convergent relationships by the similarity of active sites.

## Availability and requirements

• **Project name**: SAMO

• **Project home page**:  or 

• **Operating system(s)**: Windows, Linux

• **Programming language**: C++

• **Other requirements**: None

• **License**: FreeBSD

• **Any restrictions to use by non-academics**: licence needed

## Abbreviations

PDB: protein data bank

RMSD: root mean square distance

3D: three dimensional

LP: linear programming

SAMO: protein Structure Alignment tool based on Multiple Objective optimization

LSS: weighted Least Square Subproblem

LPS: integer Linear Programming Subproblem

## Authors' contributions

LC proposed the main idea. LYW and YW designed and implemented the algorithm. SZ and XSZ improved the model and gave valuable suggestions. All authors write and approve the manuscript.

## Supplementary Material

Additional file 1 A text file contains a brief introduction of the software. Click here for file

Additional file 2 The windows version of the software. Click here for file

Additional file 3 The Linux version of the software. Click here for file

## Figures and Tables

**Table 1:** Comparisons of structure alignment algorithms with *rms *distance and the number of aligned atoms *m*.

	Protein Pairs	SAMO			Dali		CE		Lund	
		*rms*	*m*	*λ*	*rms*	*m*	*rms*	*m*	*rms*	*m*
Reductases	1DHF_*a *_- 8DFR	0.7	182	6.0	0.7	182	0.7	182	0.7	182
	1DHF_*a *_- 4DFR_*a*_	1.8	156	6.0	2.0	154	2.0	154	2.0	156
	1DHF_*a *_- 3DFR	1.6	159	6.0	1.7	158	1.7	158	1.7	159
	8DFR - 4DFR_*a*_	1.9	157	6.0	2.0	155	2.0	155	2.0	157
	8DFR - 3DFR	1.6	159	6.0	1.8	159	1.8	158	1.8	160
	4DFR_*a *_- 3DFR	1.5	156	6.0	1.5	154	1.5	155	1.5	155

Globins	2HHB_*a *_- 2HHB_*b*_	1.4	139	6.0	1.4	138	1.5	139	1.4	139
	2HHB_*a *_- 1MBD	1.5	141	6.0	1.5	139	1.6	141	1.5	141
	2HHB_*a *_- 2HBG	1.6	140	6.0	1.7	138	1.7	136	1.6	138
	2HHB_*a *_- 1ECD	2.2	131	6.0	2.3	129	2.6	128	2.3	130
	1MBD - 2HBG	2.0	141	8.0	2.2	140	2.1	140	2.0	140
	2HHB_*b *_- 1MBD	1.6	145	6.0	1.6	145	1.6	144	1.6	145
	2HHB_*b *_- 2HBG	1.7	137	6.0	2.0	135	1.9	134	1.7	136
	2HHB_*b *_- 1MBA	2.2	140	6.0	2.3	138	2.4	139	2.4	140
	2LHB - 1MBD	1.4	137	6.0	1.4	135	1.6	137	1.5	137
	2LHB - 2HBG	1.9	133	6.0	2.0	128	2.1	130	2.0	132
	1MBD - 1MBA	1.9	143	6.0	1.9	142	1.8	141	1.9	143
	1MBA - 1ECD	1.9	136	8.0	1.9	133	2.0	134	2.0	136
	2HBG - 1ECD	2.4	129	6.5	2.6	129	2.6	125	2.5	129

Ten 'difficult' structures	1FXI_*a *_- 1UBQ	2.5	70	6.0	2.6	60	3.8	64	2.6	63
	1TEN - 3HHR_*b*_	1.7	87	6.0	1.9	86	1.9	87	1.8	87
	3HLA_*b *_- 2RHE	2.9	87	6.0	3.0	75	3.4	84	3.3	83
	2AZA_*a *_- 1PAZ	2.5	82	4.5	2.5	81	2.9	84	2.4	83
	1CEW_*i *_- lMOL_*a*_	2.3	83	6.5	2.3	81	2.3	81	2.2	82
	1CID - 2RHE	2.3	98	6.5	3.2	97	2.9	97	2.5	97
	1CRL - 1EDE	3.1	281	6.0	3.5	211	3.8	219	5.0	126
	2SIM - 1NSB_*a*_	2.9	322	6.0	3.3	291	3.0	275	2.9	292
	1BGE_*b *_- 2GMF_*a*_	3.3	110	7.5	3.3	94	3.9	107	3.3	104
	1TIE - 4FGF	2.4	115	6.0	3.1	114	2.9	116	2.7	115

Different folds	1NSB_*a *_- 1TIE	3.1	156	6.0	-		6.4	88	-	
	1NSB_*a *_- 4FGF	3.0	118	6.0	-		5.8	72	-	
	1FXI_*a *_- 1CEW_*i*_	2.9	56	6.0	-		7.2	56	-	
	1FXI_*a *_- 1MOL_*a*_	2.9	70	6.0	-		5.8	48	-	

Different Classes	1BGE_*b *_- 1TEN	2.8	82	6.0	-		7.4	40	-	
	1BGE_*b *_- 1PAZ	3.2	103	6.0	-		6.2	48	-	
	2GMF_*a *_- 1TEN	3.0	68	6.0	-		4.8	40	-	

Circular permutation	1LED - 1NLS	1.1	213	6.0	1.9	119	1.1	112	-	
	2PIA - 1AXJ	3.3	118	6.0	3.49	36	3.3	62	-	

**Table 2:** Comparisons of our algorithm with method of [24] for circularly permuted proteins.

	ID/Size	ID/Size	SAMO			Method in [24]	
			*λ*	*rms*	*m*	*rms*	*m*
Naturally occurring	1RIN/180	2CNA/237	6.0	1.581	174	0.877	45
	1RSY/121	1QAS/123	6.0	1.741	118	1.107	44
	1NKL/78	1QDM/74	6.0	2.852	72	1.823	48
	1ONR/316	1FBA/360	6.0	3.016	244	2.444	77
	1AQI/382	1BOO/282	6.0	3.329	200	3.571	66

Human made	1AVD/123	1SWG/112	6.0	2.499	98	0.815	66
	1GBG/214	1AJK/212	6.0	2.879	182	0.347	110

**Table 3:** Comparisons of our algorithm with method of [14].

ID/Size	ID/Size	SAMO			Method in [14]		
		Score	*rms*	*m*	Score	*rms*	*m*
1CHO/238	1CHO/238	1.00	0.00	238	1.00	0. 00	238
1CHO/238	2CHA/236	0.99	0.55	236	0.99	0. 55	236
1CHO/238	2PTCE/223	0.85	0.85	212	0.84	0.85	211
1CHO/238	1TPO/223	0.82	0.80	208	0.81	0. 81	207
1CHO/238	1TGSE/225	0.78	0.88	203	0.78	0.89	203
1CHO/238	2PRK/279	0.25	1.77	104	0.25	1. 67	103
1CHO/238	3ACT/218	0.26	1.69	93	0.25	1.69	86
1CHO/238	1SBT/275	0.25	1.87	111	0.24	1. 76	96
1CHO/238	2SECE/274	0.27	1.90	117	0.23	1. 78	95
1CHO/238	1FX1/147	0.23	1.94	80	0.23	1.69	71
1CHO/238	1CSE/274	0.26	1.89	117	0.23	1.72	94
1CHO/238	1TECE/279	0.27	1.94	105	0.22	1.79	94
1CHO/238	9PAP/212	0.29	1.99	102	0.22	1. 74	81
